# Who Gets Prompt Access to Artemisinin-Based Combination Therapy? A Prospective Community-Based Study in Children from Rural Kilosa, Tanzania

**DOI:** 10.1371/journal.pone.0012104

**Published:** 2010-08-10

**Authors:** Daudi O. Simba, Marian Warsame, Deodatus Kakoko, Zakayo Mrango, Goran Tomson, Zul Premji, Max Petzold

**Affiliations:** 1 Department of Community Health, Muhimbili University of Health and Allied Sciences, Dar es Salaam, Tanzania; 2 Division of International Health [IHCAR], Department of Public Health Sciences, Karolinska Institutet, Stockholm, Sweden; 3 Department of Behavioural Sciences, Muhimbili University of Health and Allied Sciences, Dar es Salaam, Tanzania; 4 National Institute for Medical Research, Kilosa Station, Tanzania; 5 Medical Management Centre, Karolinska Institutet, Stockholm, Sweden; 6 Department of Parasitology and Medical Entomology, Muhimbili University of Health and Allied Sciences, Dar es Salaam, Tanzania; 7 Nordic School of Public Health, Göteborg, Sweden; Kenya Medical Research Institute, Kenya

## Abstract

**Background:**

Effective and timely case management remains one of the fundamental pillars for control of malaria. Tanzania introduced artemisinin-combination therapy [ACT] for uncomplicated malaria; however, the policy change is challenged by limited availability of ACTs due to high cost. This study aimed to determine factors influencing prompt access to ACTs among febrile children in rural Kilosa, Tanzania.

**Methods and Findings:**

In a community-based study, 1,235 randomly selected children under five were followed up weekly for six months, in 2008. Using a structured questionnaire, children's caretakers were asked about the child's febrile history in the last seven days, and treatment actions including timing, medicines used and source of care. Caretakers' knowledge about malaria and socioeconomic and demographic data were also obtained. About half of followed-up children had at least one episode of fever. Less than half (44.8%) of febrile children were taken to government facilities. Almost one-third (37.6%; 95% CI 33.1–42.1) of febrile children had prompt access to ACT. Care-seeking from a government facility was the overriding factor, increasing the likelihood of prompt access to an ACT 18 times (OR 17.7; 95% CI 10.55–29.54; adjusted OR 16.9; 95% CI 10.06–28.28). Caretakers from the better-off household (3rd–5th quintiles) were more likely to seek care from government facilities (OR 3.66; 95% CI 2.56–5.24; adjusted OR 1.80; 95% CI 1.18–2.76). The majority of antimalarials accessed by the poor were ineffective [86.0%; 295/343], however, they paid more for them (median Tsh 500) compared to the better-offs (median Tsh 0).

**Conclusions:**

Prompt access to ACT among febrile children was unacceptably low, due mainly to limited availability of subsidised ACT at the location where most caretakers sought care. There is urgent need to accelerate implementation of strategies that will ensure availability of ACT at an affordable price in remote rural areas, where the burden of malaria is highest.

## Introduction

Effective malaria case management, that is, early diagnosis and prompt treatment of malaria patients, remain to be one of the fundamental pillars of malaria control measures. The current advocated global targets include achievement, by 2010, of universal coverage of all malaria patients diagnosed and treated with effective antimalarial medicines within one day of the onset of illness [1]. Additionally, the target of the Roll Back Malaria [1] Partnership is to have, by 2010, at least 80% of children underfives receiving effective treatment with an ACT, within 24 hours of the onset of symptoms. The implementation of such policy change is not without challenges, and hence, research is an important tool to better understand barriers and best practices [2]. Several studies have been conducted across Africa, following the introduction of ACT policy [3], [4], mostly evaluating implementation of the policy change at facility level, and specifically on the availability of drugs and the clinicians' prescribing habits. Very few studies have explored the implications of policy change for access to treatment in the community [5], [6]. Notwithstanding, a study conducted by Gitonga et al in Kenya reported that only 10% of the children with fever had access to ACT within 48 hours, while Tipke et al reported no children in Burkina Faso gained access despite the policy change [5], [6].

In Tanzania, malaria which is the leading cause of morbidity and mortality accounts for over 40% of out-patient attendances and 36% of all death in under fives [National Malaria Control Programme - Medium term malaria strategic plan, 2008–2013]. Tanzania accommodated the malaria treatment policy change for uncomplicated malaria from sulphadoxine-pyrimethamine (SP) to ACT in October 2006 [Ministry of health and social welfare, National guideline for malaria diagnosis and treatment, 2006]. However, procurement of ACT is mainly through the support of funding from the Global Fund to Fight AIDS, Tuberculosis and Malaria and caters for ACT provision by government and not-for-profit private health facilities, which include Faith Based Organisation [FBO] facilities. In the former, free services are provided to under-fives, whereas in the latter, a considerable fee is charged, even if the ACT is free. Furthermore, most treatment for malaria is provided outside the formal healthcare sector, usually by shopkeepers where treatment is often inappropriate [7]. Recently, the country introduced an ‘Accredited Drug Dispensing Outlet’ (ADDO) system in which drug stores that normally sell non-prescription medications are licensed to sell a selected number of prescription-only pharmaceuticals, including the recommended ACT, that is, artemether-lumefantrine (ALu). Tanzania is one of the countries invited by the Affordable Medicine Facility for malaria [AMFm] to implement a new global health initiative that aims to lower ACT prices [8] that will benefit even the private sector.

A recent Malaria Indicator Surveys (MIS) conducted in Tanzania revealed that only 13% of the febrile children had prompt access to the ACT [National Malaria Control Programme, 2009: Summary of five household surveys to monitor population-level coverage and impact of malaria interventions in Tanzania, 2007–08]. Studies conducted prior to ACT era indicated availability of medicines, source of care, perceptions about causes and the severity of illness, the efficacy of treatment, the ability to pay, and the distance to the health facility as factors influencing timely access to malaria treatment [9], [10]. Normative data on factors influencing the prompt access to ACT are necessary to inform implementation of drug policy change [7], [11]. Therefore, this study was conducted in order to determine the magnitude of, and identify factors influencing prompt access to ACT among under-five children in rural Kilosa, Tanzania.

## Materials and Methods

### Study area

Kilosa district is situated about 300 kilometres west of Dar es Salaam, the largest business city in Tanzania. The district has about 488,000 people, of which 79,000 are under five years [National Census, 2003]. Administratively, the district has seven divisions with 161 registered villages and is divided into four geographic-climatic areas: wet-highland, dry-highland, wet-lowland and dry-lowland [District annual report, 2002]. The district has an under-five mortality of 166 per 1,000, and about 32% of the people live below the poverty line [District Medical Officer's Report, 2006]. The main sources of income are farming and livestock keeping. Food crops produced at subsistence level are maize (staple food), cassava, rice and sorghum. In 2007, malaria accounted for more than half [55.5%] of the total outpatient attendance, and 60% of the total deaths among under-fives admitted to the hospitals [District Annual Report, 2008]. Malaria transmission in the wet-lowland area occurs throughout the year, with the highest incidence during and soon after the rain season, March to June.

Kilosa has 71 health facilities, comprised of 3 hospitals, 7 health centres and 61 dispensaries [District Annual Report, 2008]. Of these, two hospitals, one health centre and nine dispensaries belong to FBOs, and charge a fee for consultation, drugs and laboratory tests. In the public facilities, services for under-fives are offered free of charge. There were 152 drug shops in the district, most of them found in urban and semi-urban areas. At the time of the study, about 92 drug shops met the minimum criteria set by the Tanzania Food and Drug Agency (TFDA) and were upgraded to the status of ADDO, which allowed them to sell artemether-lumefantrine (ALu). ADDOs are able to purchase ALu at a subsidised price and charge a flat rate of US $0.50 for one paediatric treatment course [Stakeholders' Meeting Report on ADDO Program, Ministry of Health and Social Welfare, July 2008].

### Study design sample size and selection

This is a prospective study design, in which 1,200 randomly selected children aged 3–59 months were followed up at their homes weekly for six months, in 2008. Weekly follow ups were chosen so as to maximize recall of events. The outcome measure was prompt access to ACT among children who had fever episode as reported by the caretaker. Fever was used as a proxy for malaria given the absence in rural areas of laboratory facilities for diagnosing malaria and the national treatment guidelines that advised treating childhood fevers suspected to be malaria with the recommended first-line treatment. This proved for us to be of practical use as reported by other researchers [5], [6], [9].

Stratified random sampling was performed by dividing rural villages into the four geographic-climatic areas, wet-highland, dry-highland, wet-lowland and dry-lowland, using a list of villages from the national census. In each area, one village was randomly selected from among villages with health facilities, and two from those without, making a total of 12 villages. Under-five children were randomly selected from village registers. Village registers are kept by the Village Executive Officer in all villages within the country. However, in many villages these registers were not up-to-date. Therefore, village registers had to be updated by the researchers with the assistance from the village health workers (VHWs) via house-to-house visits. A sample size of 1,200 was thereby apportioned to the 12 villages, according to village population size and random selection was performed using EPI-INFO, software (version 6). The sample size was derived by requiring a maximum length of 95% confidence interval +/−5% units, and assumed a proportion of 50%. Adding 10% for possible loss to follow-up, and 38% correction factor for clustering effect on village level [ICC = 0.01], the total sample size came to 583 (approximated to 600). Available data reported by patients/caretakers indicated that the 2-week period incidence of fever suspected to be due to malaria in Tanzania was 6% [12]. Assuming, 6 out of 100 children developed fever in a 2-week period, we needed to follow-up 1,200 children for 16 weeks in order to attain a sample size of 600. ACT is not recommended in very young children (according to manufacturer's instructions) and, therefore, only children aged 3–59 months with caretakers who were residents for at least six months were enrolled.

### Data collection procedure

Two to four VHWs were recruited in each village, depending on the village size. These were centrally trained for six days on the purpose of the study, data collection procedures and ethical issues. Three research assistants [13], each responsible to four villages, were recruited to supervise the VHWs.

Baseline and follow-up questionnaires were developed based on previous literature [12], [14] and were refined after a 2-week pilot-test in two of the villages. At enrolment of the study children, RAs interviewed caretakers at home. Using a structured-questionnaire, data were collected on demographic characteristics, socioeconomic status [SES] and knowledge about malaria. Thereafter, VHWs followed up enrolled children every Saturday and Sunday to find those who had developed fever in the week preceding, and also interviewed caretakers using the structured questionnaire. During the interviews, fever episode were recorded and included other accompanying symptoms, where care was sought and the type of treatment obtained. The date and time (categorised as morning, afternoon and night) of fever onset and the administration of ALu (*dawa mseto*) were obtained from caretaker's report. Children, who had not recovered at the time of the interview, completed their information on what happened after the previous encounter during the next round. Children found to have severe symptoms on the day of interview were advised to be taken immediately to the health facility.

In government health facilities, medical notes are written in notebooks and are kept by caretakers. These, as well as drug containers, were examined to verify the type of drug provided. Where such evidence was not available, verification was performed by displaying samples of locally available brands [15]. Distance was estimated by the RAs by charting mileage to the nearest governmental facility by using motorcycles. On each Monday, meetings were held with VHWs and RAs, where forms were collected and scrutinised for accuracy.

### Data analysis

Data were double entered into EpiData 3.0 [EpiData Association. Odense, Denmark] and analysed using STATA version 10.1 [Stata Corp., College station, TX, USA]. The outcome measure was ‘prompt access to ACT’ which was defined as treatment with any type of ACT on the same or the next day of fever onset [5], [10]. Dates were used instead of hours in calculating promptness because of difficulties in reporting the time in communities where the majority of people do not have watches [10]. The outcome variable was binary, coded as child received the ACT or not. Since no child had the ACT from more than one source, no confusion arose from seeking care through multiple sources. Analysis was based on the first fever episodes. Since each child had at least one episode, each first episode represented a child. This was done in order to ensure equal representation of the study subjects and to avoid within-child dependencies. Separate analysis for episode 2 to 6 could not be done due to small sample size. In addition, the predictors for a child's access to ACT would hardly change over a short time period of six months. SES was calculated using Principal Component Analysis in which 19 assets adopted from the Tanzania Demographic Health Survey were used [16]. Variables that had 90% or more of the study children in one category were excluded. The final list included material composing the floor, walls and the roof; the source of drinking water and type of light; and ownership of a mattress, an iron, a bednet, a bicycle and a sofa. The first component explained 37.4% of the variability. SES index was categorised in five quintiles. Households in the first and second quintiles were classified as poor and the others as better-offs, after initial analysis showed no statistically significant association of each quintile with the outcome variable. We also collected data on the cost of antimalarials and other drugs, as well as other services such as laboratory services, and expenditure resulting from seeking care. The latter included, for example, hiring a bicycle and buying food on the way. However, we conducted analysis based on the expenditure for drugs because of inconsistencies in reporting other items. The number of under-fives and the total number of people residing in a household were also recorded. The hypothesis was that many under-fives might pose a burden to the caretaker, in carrying them to the facilities if no one is at home; while small families give no option for the caretaker to leave the household chores to someone else.

Bivariate analysis was performed on variables likely to influence the prompt access to the ACT as well as the source of care. All variables that were found to have a statistically significant association were introduced into the multiple regression models using stepwise analysis, with a cut-off point of 0.10. All regression analysis were weighted. Post-stratification weighting was done at the strata level (wet highland, dry highland, wet lowland, dry lowland) after observing that the sampling proportion did not perfectly match the true distribution of the population. Odds ratios [OR] with 95% confidence intervals [CI] were used to measure association. Significance was tested at the 5% level. Variables introduced in the multiple regression model to determine an association of prompt access to the ACT were the total number of people in the household, number of under fives, the source of care, severe symptoms (loss of consciousness or ‘malaria had gone to the head’, cerebral malaria), separate individual symptoms, SES, knowledge on the recommended drug for the treatment of uncomplicated malaria (*malaria ya kawaida*) and knowledge about the causes of convulsion to children. The total number of people in the household and all individual symptoms except cough were found not statistically significant. The rest of the variables were introduced in a stepwise analysis multiple regression model where only the source of care and knowledge on the recommended drug for the treatment of uncomplicated malaria were retained in the final model. In determining factors influencing care-seeking from government health facilities the same variables used in the previous model were analysed. Failure to feed, cough, SES and knowledge of treatment for uncomplicated malaria and knowledge about the cause of convulsions were found to be statistically significant. These were introduced in a stepwise regression model and all variables except cough and knowledge on cause of convulsion were retained in the final model.

### Ethical clearance

Ethical clearance was obtained from the Muhimbili University of Health and Allied Sciences and the National Institute for Medical Research (NIMR), Tanzania. Permission to conduct the study was also obtained from the regional and district authorities. A written informed consent was obtained from each adult caretaker of the child during baseline data collection.

## Results

During the six-month collection period, 35 children were added to the enrolment after children, who had either attained the age of 60 months or who were reported to have migrated during the study period, were replaced by random selection. In all, 1,235 children aged 3–59 months were enrolled. There was no difference between febrile and non-febrile children in relation to the socio-demographic characteristics of the children and their caretakers (Table 1). The median age of children who had fever was 28.0 months, and that of their caretakers was 28 years. In total, 957 episodes of fever were recorded from 607 children, ranging between one and six episodes per child.

**Table 1:** Background characteristics of study population by reported fever status and their caretakers.

Characteristics of parent and child	Reported to have had no fever [n = 628]		Reported to have had fever[n = 607]		Total
	Number	*%*	Number	*%*	*Number*
*Caretaker's age*					
<30 year	358	51.5	337	48.5	695
30+ years	270	50.0	270	50.0	540
*Child's age*					
<2 years	227	44.9	279	55.1	506
2+ years	401	55.0	328	45.0	729
*Child's sex*					
Male	319	50.2	316	49.8	635
Female	309	51.5	291	48.5	600
*Socio-economic status (quintiles)*					
1 Lowest	162	56.1	127	43.9	289
2 Second	130	54.6	108	45.4	238
3 Middle	117	54.7	97	45.3	214
4 Fourth	118	47.4	131	52.6	249
5 Highest	101	41.2	144	58.8	245
*Marital status*					
Not married	229	52.1	211	47.9	440
Married	399	50.2	396	49.8	795
*Education in years*					
<7 years	244	52.3	223	47.7	467
7+ years	384	50.0	384	50.0	768
*Distance to government facility*					
5+ km	337	58.1	243	41.9	580
<5 km	291	44.4	364	55.6	655

### Source of care

**Figure 1:**
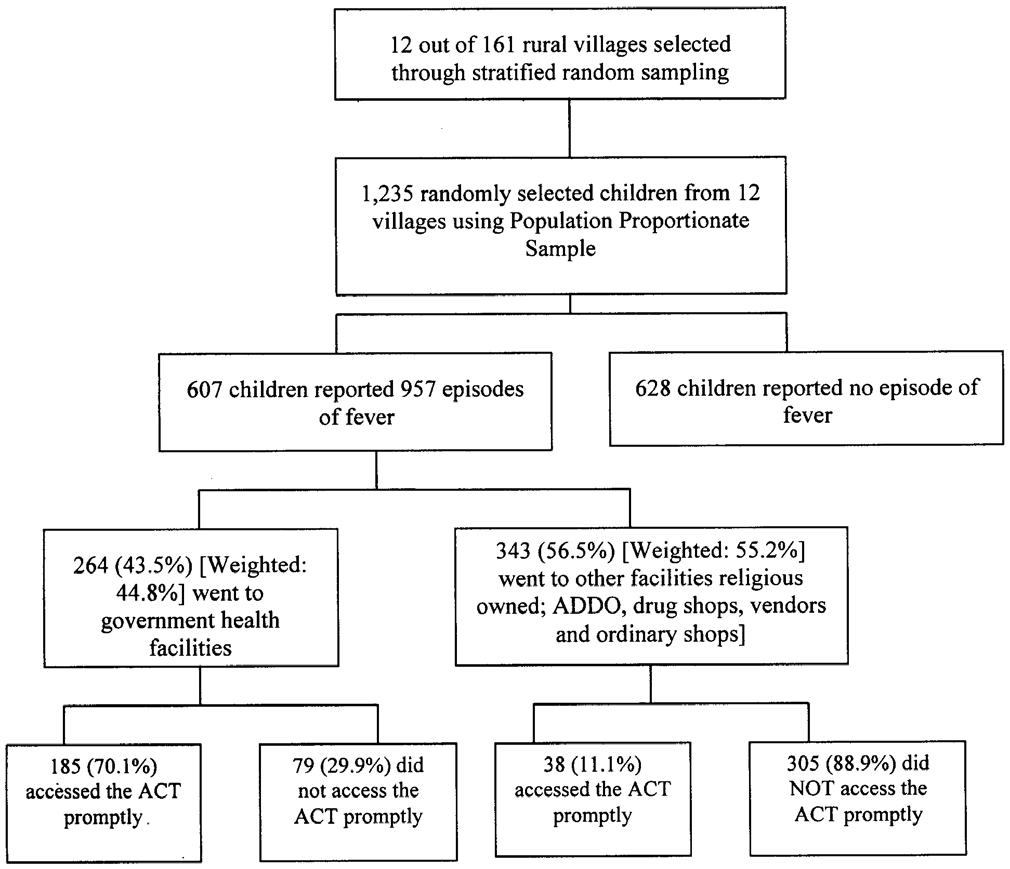
Flowchart showing the number of enrolled children by type of provider sought, treatment given and promptness of access to artemisinin combined therapy.

Out of the 607 children with fever, 264 (44.8%; 95% CI 40.3–49.4) were taken to government health facilities for care in the course of their illness (Figure 1). No caretaker sought care from the same source twice and a child who was taken to a government facility, whether as first or second option, was regarded as having gone to a government health facility. Those who were never taken to government health facilities, nearly half (44.6%; 153/343) were taken to ordinary shops, 21.6% (74/343) to FBO facilities, 15.5% (53/343) to drug shops or ADDOs, 12.8% (44/343) had home care, 5.0% (17/343) went to traditional healers, and 0.6% (2/343) to private facilities.

### Type of treatment obtained and costs

Of the 607 children, 374 (weighted: 66.3%; 95% CI 62.2–70.4) received antimalarials, of which, 268 (45.8%; 95% CI 41.2–50.5%) were ACT, and all were ALu. The majority of those who were taken to government health facilities were treated with ALu (83.3%; 220/264), while only 14.0% (48/343) of those who were taken to the other facilities received ALu. Children from the better-off household (3^rd^–5^th^ quintiles) were more likely to receive ALu (52.4%; 195/372) compared to the poor (31.1%; 73/235), (p-value<0.001).

The average expenditure on drugs reported by caretakers was Tanzanian shillings (Tsh) 767 (95% CI; Tsh 633–902), and the median was Tsh 200 (1,200 Tsh equivalent to US dollar 1). The median expenditure on drugs at government health facilities was zero. Within the private sector, the median expenditure on drugs was highest among FBO facilities (Tsh 2,500) followed by ADDO (Tsh 1,600) and drug shops (Tsh 900). Median expenditure was least at ordinary shops (Tsh 500). Caretakers from the poor households paid more compared to the better-offs, median expenditure Tsh 500 and zero, respectively.

### Factors influencing promptness of access to ACT

Overall, 223 of 607 (37.6%; 95% CI 33.1–42.1) of the children who had fever received ALu promptly. Prompt access to the ACT was higher (70.1%; 185/264) among children taken to the government health facilities compared to those taken elsewhere (11.1%; 38/343), see Figure 1. Prompt access to the ACT at FBO facilities and ADDOs were 31.4% (27/86) and 21.4% (6/28), respectively.

Seeking care from a government health facility and knowledge that d*awa mseto* (Coartem®) is the recommended drug for the treatment of *malaria* ya *kawaida* [uncomplicated malaria] influenced prompt access to ACT. Together, the two factors explained for 30% of the variability. Table 2 shows that the odds of getting prompt access to the ACT was about 18-times higher among children seeking care from government health facilities compared to those who were taken elsewhere (OR 17.7; 95% CI 10.55–29.54; adjusted OR 16.9; 95% CI 10.06–28.28). Although univariate analysis showed that children from better-off households were 2-times more likely to access the ACT promptly than the poor this effect was diluted when other variables were introduced in the regression model.

**Table 2:** Determinants of accessing appropriate antimalarials (ACT) on the same or next day (promptly) for children under five reported to have fever by geographical-climatic strata.

	Unadjusted OR		Adjusted OR	
**Prompt access to ACT in all strata** * **n = 607**	**OR**	**95% CI**	**OR**	**95% CI**
Seeking care at government health facility	17.7	10.55–29.54	16.87	10.06–28.28
Better-offs [Quintile 3+]	2.1	1.39–3.28		
Convulsion and loss of consciousness	1.9	0.57–6.26		
Knowledge that high fever or mosquitoes causes convulsion to children	0.7	0.50–1.09		
Knowledge that d*awa mseto* (Coartem®) is the recommended drug for the treatment of *malaria ya kawaida* [uncomplicated malaria]	2.04	1.36–3.07	1.26	0.76–2.09
**Prompt access to ACT in Wet-Lowland areas n = 174**				
Seeking care at government health facility	19.4	6.5–57.5	16.77	6.55–42.97
Better-offs [Quintile 3+]	0.4	0.1–1.4		
Convulsion and loss of consciousness	3.1	0.60–16.4		
Knowledge that high fever or mosquitoes causes convulsion to children	0.7	0.34–1.61		
Knowledge that *dawa mseto* (Coartem®) is the recommended drug for the treatment of *malaria ya kawaida* [uncomplicated malaria]	10.8	2.6–44.6	7.76	1.54–39.08
**Prompt access to ACT in Dry-Lowland areas n = 173**				
Seeking care at government health facility	23.9	9.1–62.8	17.75	7.10–44.37
Better-offs [Quintile 3+]	0.8	0.3–2.2		
Convulsion and loss of consciousness**	-	-		
Knowledge that high fever or mosquitoes causes convulsion to children	0.6	0.28–1.18		
Knowledge that *dawa mseto* (Coartem®) is the recommended drug for the treatment of malaria ya kawaida [uncomplicated malaria]	0.7	0.3–1.9		
**Prompt access to ACT in Wet-Highland areas n = 158**				
Seeking care at government health facility	3.4	1.0–11.6	3.91	1.33–11.47
Better-offs [Quintile 3+]	1.3	0.4–3.5		
Convulsion and loss of consciousness	3.85	0.59–25.1		
Knowledge that high fever or mosquitoes causes convulsion to children	1.3	0.54–3.2		
Knowledge that *dawa mseto* (Coartem®) is the recommended drug for the treatment of malaria *ya kawaida* [uncomplicated malaria]	1.7	0.6–5.3		
**Prompt access to ACT in Dry-Highland areas n = 102**				
Seeking care at government health facility	43.5	8.7–216.7	38.34	10.44–140.73
Better-offs [Quintile 3+]	0.6	0.2–2.2		
Convulsion and loss of consciousness	2.18	0.13–36.0		
Knowledge that high fever or mosquitoes causes convulsion to children	3.0	1.29–7.14	2.43	0.77–7.71
Knowledge that *dawa mseto* (Coartem®) is the recommended drug for the treatment of *malaria ya kawaida* [uncomplicated malaria]	3.0	0.8–11.0		

*Weighted.

**Not computed due to inadequate data.

Further analysis by geographic-climatic strata showed a similar pattern with regard to the source of care, except in the wet-highland stratum where the association was less remarkable (OR 3.91; 95% CI 1.33–11.47) (Table 2). In the wet-lowland area, children whose caretakers were knowledgeable of the recommended drug for the treatment of uncomplicated malaria (*malaria ya kawaida*) were 8-times more likely to access the ACT promptly (OR 10.8; 95% CI 2.6–44.6, adjusted OR 7.76; 95% CI 1.54–39.08) compared to those who did not have the knowledge, see Table 2.

### Factors influencing seeking care from government health facilities

Since seeking care from a government health facility was the main factor associated with prompt access to the ACT, further analyses was done to determine factors that influence seeking care from these facilities. Table 3 shows that caretakers who knew that ALu (*Dawa mseto*) is the recommended drug for the treatment of uncomplicated malaria (*malaria ya kawaida*), were more likely to take their children to a government health facility compared to those who did not know (OR 2.55; 95% CI 1.71–3.81; adjusted OR 2.06; 95% CI 1.31–3.25). Knowledge about mosquitoes, and that high fever causes convulsion in children, increased the likelihood of taking a child to a government health facility in dry-highland areas; however, the difference was not significant (OR 3.0; 95% CI 1.29–7.14; adjusted OR 2.43; 95% CI 0.77–7.71).

**Table 3:** Determinants of seeking care from government facilities for children under five reported to have fever in Kilosa district.

Determinants for seeking care from government HFs in all strata*	Unadjusted		Adjusted	
	OR	95% CI	OR	95% CI
Having only one under five in the household	1.42	0.96–2.09		
Using own or hired bicycle to go to health facility	2.04	1.15–3.60		
Not feeding	2.01	1.20–3.34	1.85	1.06–3.22
Cough	0.67	0.45–0.98	0.75	0.49–1.14
Better-off (3–5 quintiles )	3.22	2.10–4.92	2.56	1.63–4.01
Knowledge that high fever or malaria causes convulsion to children	0.74	0.51–1.084		
Knowledge that *dawa mseto* (Coartem®) is the recommended drug for the treatment of *malaria ya kawaida* [uncomplicated malaria]	2.55	1.71–3.81	2.07	1.35–3.16

*weighted.

n = 607.

Children from the better-off households were twice more likely to be taken to a government health facility compared to those from poor households (OR 3.22; 95% CI 2.10–4.92; adjusted OR 1.89; 95% CI 1.19–3.03). Moreover, the better-offs were found to live closer to government health facilities (weighted median, 2 km) compared to the poor (weighted median, 5 km). Further analysis showed that the better-offs were 4-times more likely to have knowledge on the recommended drug for treatment of uncomplicated malaria (OR 3.74; 95% CI 2.65–5.28), and that they were more likely (2.5-times) to own radios compared to the poor (OR 2.35; 95% CI 1.68–3.30).

## Discussion

This study shows that nearly two-third of the children with fever received antimalarials on the same or next day of the onset of fever, of which less than half were ALu. Furthermore, only one-third of the children had prompt access to the ACT, and that seeking care from government health facilities was the major determining factor. The study has also shown that, despite subsidy on ACT, the poor were paying more, as compared to the better-offs, for the ineffective drugs they got from non-government facilities simply because they had less access to government health facilities. Previous studies have also reported source of care as one of several factors influencing access to antimalarials [9], [10]. However, it was an overriding factor influencing access to prompt ACT in this study. The main difference between this study and others is that, previous studies were conducted when chloroquine (CQ) or SP were the recommended first-line drugs which could be obtained easily and at a very cheap price, even in ordinary shops [17], [18], [19], [20]. The current study is the first to report on factors influencing access to ACT, and underscores the fact that, when it comes to accessing an expensive drug such as ACT, in a setting where subsidy is limited to some facilities, the source of the care becomes the main determinant to prompt access.

Encouragingly, prompt access to the ACT was very high among children taken to government health facilities, in this study. However, the fact that less than half of these children were taken there, raises some pertinent questions as to why most caretakers did not seek care from government health facilities where the ACT was provided free of charge. This can be explained by the findings from previous studies that reported caretakers residing closer to a government health facility were more likely to seek care from these facilities [21], [22]. The present study found that the better-offs were living closer to government health facilities and had better knowledge on the recommended treatment compared to the poor.

The majority of caretakers who did not seek care from government health facilities were from the poor households, this confirms the ‘inverse care law’ stating that “the availability of good medical care tends to vary inversely with the need for the population served” [23]. Unlike with previous studies where the better-offs were reported to access the recommended antimalarials by paying more in non-government facilities [24], in the current study, it was the poor who paid more for the inappropriate drugs bought at non-government facilities. This irony serves as another evidence of the uniqueness of ACT as an effective antimalarial, but with limited subsidy that ends up marginalising the poor.

Subsidy has made ALu quite cheaper thus affordable to many people. But despite the subsidy, prompt access to the drug was very low among children taken to FBO facilities and ADDOs. Most of the FBO facilities are situated in rural remote areas and serve poor communities. Unfortunately, they charge a considerable fee to cover operating costs [25]. This could explain the small proportion of the febrile children taken to these facilities. Thus, even if ALu was provided for free, payments for other drugs, consultation fees and laboratory services would still act as a deterrent. In a community where almost one-third of the population lives on less than a dollar per day [District Medical Officer's Report, 2006], the average of two US dollars charged by FBO facilities on drugs cannot be afforded by many. This calls for a need to examine how FBO facilities can be mobilised in the strategy to improve prompt access to the ACT, especially, in rural remote areas. Low utilization of ADDOs might be explained by the fact these facilities, as well as drug shops, are mostly situated in urban and semi-urban areas [26]. Hence, the dramatic rise in the purchase of ACT, reported in the study by Sabot et al (2009), might not necessarily translate into equitable improvement in access to ACT in the community [27].

Knowledge that ALu is the recommended drug for the treatment of uncomplicated malaria was associated with increased prompt access to the ACT in the wet-lowland areas, where malaria transmission is high and occurs throughout the year. This might be an indication of the successful advocacy campaign by the National Malaria Control Programme. Radio and television adverts and programmes featuring ALu as the recommended first-line drug have been aired since the inception of the new drug policy. Such messages might have been taken more seriously in areas where malaria transmission is intense. The lack of association between SES and prompt access to the ACT found in this study might be due to the overwhelming influence of the source of care which might have concealed the influence of poverty on prompt access.

### Methodological considerations

Diagnosis of malaria relied on caretaker's report of fever. This might result in underestimation of prompt access to the ACT since not all fevers are due to malaria. However, the fact that Kilosa is hyperendemic area increases the likelihood of most fevers to be due malaria. Underestimation of prompt access might also have resulted from including children who were suspected to have severe malaria and were correctly treated with the second line antimalarials, or from children who were found to have a negative blood slide for malaria parasites and were treated with other drugs [28]. The current WHO treatment policy recommends prompt parasitological confirmation by microscopy or rapid diagnostic tests, where available [29]. However, findings from this study, based on fever as malaria, are still relevant in many areas where such policy is yet to be fully implemented, and at all levels of service provision, including the private for and not-for-profit sector.

Ascertainment of the drug given to a child can be difficult, especially if the caretaker does not know how to read and write. Therefore samples of the drug packets and records from the clinician or drug shop were used to verify the type of drug given [5], [15]. Analysis was limited to drug costs due to simplicity in collecting the data and inconsistencies in reporting other expenditure items by caretakers.

The use of village health workers with low level of basic education might have undermined data quality. However, given the one-week training prior to data collection, the day-to-day close supervision by RAs and the weekly meetings, the problem of data quality might have been minimized. Further, the presence of village health workers might have caused a ‘Hawthorn effect’ likely to overstate the level of prompt access to the ACT.

Findings from strata analysis should be interpreted with caution because the sample size for this study was calculated to power the overall analysis and not the strata specific analysis.

### Conclusions

The major drawback to promptly accessing ACTs found in this study was the limited availability of subsidized ACT in places where most caretakers took their children for care. And despite subsidy on the ACT, a majority of caretakers, especially from the poor households, continue to pay more for ineffective drugs in non-government facilities.

Government health facilities proved to be the best source for accessing prompt treatment, as judged by the highest rate of prompt access to ACTs. Therefore, greater part of the global effort on malaria should focus on strengthening the public sector. However, government health facilities were accessed mostly by the relatively wealthy. Therefore, access to government health facilities need to be improved by optimizing the location of health facilities - nearer the malaria hotspots. In the non-government sector, merely subsidizing the price of ACT is insufficient. In addition to the subsidy, the quality of services must be regulated to provide the recommended treatment. Furthermore, information must be provided effectively to the poor on the best treatment and their sources. Further research should address why prompt access to ACT in ADDOs and FBOs is low despite ACT subsidy, and how FBO facilities, located mostly in remote rural areas, can be maximally utilised to reach the marginalized children.
